# Allergic Contact Dermatitis: Immunopathology and Potential Therapeutic Strategies

**DOI:** 10.3390/jcm14207175

**Published:** 2025-10-11

**Authors:** Anders Boutrup Funch, Carsten Geisler, Charlotte Menné Bonefeld

**Affiliations:** 1 LEO Foundation Skin Immunology Research Center, Department of Immunology and Microbiology, University of Copenhagen, 2200 Copenhagen, Denmark; cge@sund.ku.dk (C.G.); cmenne@sund.ku.dk (C.M.B.); 2 Swiss Institute of Allergy and Asthma Research, University of Zurich, 7260 Davos, Switzerland

**Keywords:** allergic contact dermatitis, rapid-onset ACD, CD8^+^ T_RM_ cells, neutrophils, novel therapeutic strategies

## Abstract

Allergic contact dermatitis (ACD) is a common inflammatory skin disease induced by exposure of the skin to contact allergens. Classically, ACD is defined as a delayed-type (type IV) hypersensitivity reaction mediated by allergen-specific T cells, with symptoms peaking 48–72 h after exposure to the contact allergen. This delayed response to the contact allergen is seen during patch testing, where allergen-naïve, unaffected skin of allergic individuals is exposed to the contact allergen. However, in daily life and in certain occupational settings, allergic individuals often experience rapid flare-ups/exacerbations with intensely itching erythema, oedema, and often vesicles within hours after re-exposure to the specific contact allergen. These rapid flare-ups only develop at skin sites previously exposed to the contact allergen. Thus, it is important to distinguish between the rapid-onset reaction typically experienced by the allergic individual and the delayed-type reaction typically seen after patch testing. This review summarizes current insights into the immunopathology of rapid- versus delayed-type ACD reactions and outlines potential therapeutic opportunities, as well as their current limitations, against rapid-onset ACD, including modulation of cytokine signaling, T cell survival, checkpoint pathways, and redox balance.

## 1. Introduction

Allergic contact dermatitis (ACD) is an inflammatory skin disease that occurs in sensitized individuals upon re-exposure to the sensitizing contact allergen [1]. The condition is highly prevalent, affecting approximately 20% of the population [2]. ACD is classically known as a delayed-type or type IV hypersensitivity reaction that is mediated by allergen-specific memory T cells. ACD manifests as local erythematous, pruritic, and occasionally blistering skin lesions that typically emerge within 48–72 h after exposure to the contact allergen, as observed during patch testing [1,3,4]. However, in daily life, patients often experience much faster (<24 h) reactions, here referred to as rapid-onset ACD, when exposed to the contact allergen in allergen-experienced skin areas [5,6,7]. The accelerated ACD response is particularly relevant in occupational settings, where repeated exposure to contact allergens in the same skin area is common. Occupational exposures are recognized as a major risk factor for ACD, with notably high prevalence rates among hairdressers, healthcare workers, and individuals in industries involving reactive chemicals, where complete avoidance of allergens is highly impractical [8,9,10]. Consequently, occupational ACD has considerable socio-economic repercussions, including frequent work absences [11,12]. Given these challenges and the current limited therapeutic options available, it is essential to study the immunopathology and identify potential therapeutic strategies, especially for individuals suffering from rapid-onset ACD.

It is known that CD8^+^ skin-resident memory T (T_RM_) cells develop locally in the skin after exposure to many contact allergens, and recently it was shown that the ability of CD8^+^ T_RM_ cells to recruit massive numbers of neutrophils into the skin is crucial for rapid-onset ACD [7,13,14,15,16]. The vast majority of infiltrating neutrophils disappear from the skin less than 48 h after exposure to the allergen [13]. Furthermore, only a limited number of neutrophils are found after exposure of allergen-naïve skin to contact allergens [7,13,16,17,18], which may explain the hitherto rather limited focus on neutrophils in ACD.

Neutrophils are short-lived immune cells that play a vital role in host defense as they are rapidly recruited to infected peripheral tissues, where they effectively phagocytose pathogens and damaged cells and exert a broad range of cytolytic effector functions. [19,20]. However, when inflammation becomes excessive or misdirected, as seen in rapid-onset ACD, neutrophil recruitment and uncontrolled release of their cytolytic granules can lead to substantial tissue damage [19,20]. In recent years, the interest in the pathological role of neutrophils in both murine models of ACD [13,14,21,22,23,24,25,26,27,28] and in human studies [16,18,29] has grown.

This review summarizes the current knowledge on the interplay between CD8^+^ T cells and neutrophils in both the sensitization and challenge phases of ACD and outlines how this translates into distinct clinical manifestations of rapid-onset versus delayed-type ACD. Furthermore, we highlight the reported limitations of the current therapeutics available, underscoring the need for tailored treatment strategies targeting both classical delayed-type and rapid-onset ACD.

## 2. The Sensitization Phase and Development of CD8^+^ T_RM_ Cells

**Figure 1:**
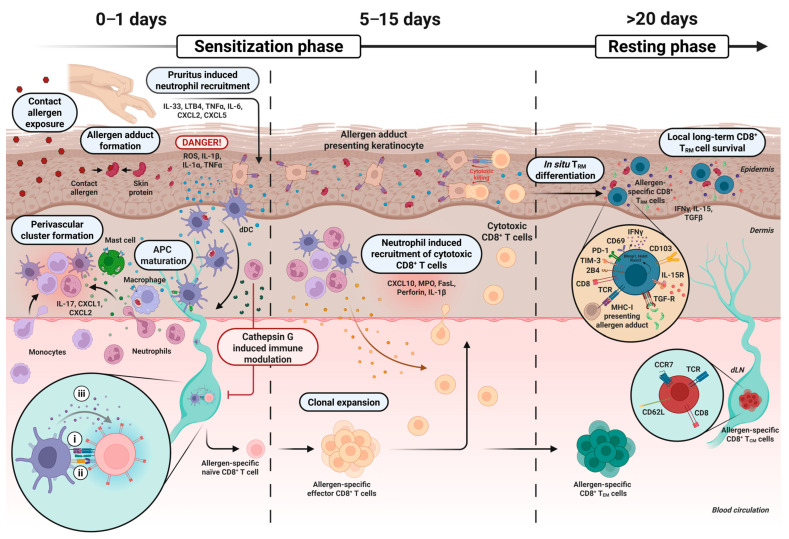
The sensitization phase and generation of epidermal CD8^+^ T_RM_ cells. Within 24 h after exposure of the skin to a contact allergen, the formation of allergen adducts occurs and skin cells initiate local danger signaling. Dermal dendritic cells (dDCs) become activated and migrate to draining lymph nodes (dLNs), where they prime naïve CD8^+^ T cells and drive clonal expansion of effector CD8^+^ T cells through three key signals: (i) the presentation of allergen-adducts via the MHC to the T cell receptor (TCR); (ii) the expression of co-stimulatory molecules; and (iii) the release of polarizing cytokines that direct T cell differentiation. In parallel, interleukin-1β (IL-1β) and other cytokines activate IL-1R-expressing mast cells and macrophages promoting neutrophil and monocyte recruitment from the circulation and the formation of perivascular immune clusters. By day 5–15, cytotoxic effector CD8^+^ T cells infiltrate the skin and kill keratinocytes that present allergen adducts. After ~20 days, allergen-specific memory T cell subsets, including CD8^+^ epidermal-resident memory T cells (T_RM_), CD8^+^ central memory T cells (T_CM_), and circulating CD8^+^ effector memory T cells (T_EM_), develop. Epidermal CD8^+^ T_RM_ cells are regulated via various inhibitory checkpoint receptors, including PD-1, TIM-3, 2B4, interleukin-15 receptor (IL-15R), and transforming growth factor-β receptor (TGF-R), and constitutively produce IFN-γ. Constitutive TCR signaling facilitates long-term survival of allergen-specific T_RM_ cells. Other abbreviations: CXCL, C-X-C motif ligand; ROS, reactive oxygen species; TNF-α, tumor necrosis factor-α; LTB4, leukotriene B4; MPO, myeloperoxidase; FasL, Fas ligand; MHC, major histocompatibility complex; TCR, T cell receptor; CCR7, C-C chemokine receptor type 7.

During sensitization, the skin is exposed to a contact allergen, which most commonly is a small (<500 Dalton) electrophilic chemical that readily penetrates the cornified barrier of the epidermis and thus come into contact with live keratinocytes and immune cells [1,30]. Once in the skin, the electrophilic properties of the allergen promote the formation of covalent bonds with the nucleophilic amino acids present in most skin proteins [30]. This results in the generation of highly stable contact allergen–self protein complexes, also referred to as allergen adducts, of which some can be deposited for months in the skin [31,32]. The allergen adducts are recognized by resident skin cells that react by producing pro-inflammatory cytokines, including interleukin (IL)-1β and tumor necrosis factor (TNF)-α, and reactive oxygen species (ROS) [33,34,35,36,37,38]. This activates antigen presenting cells (APCs) (i.e., Langerhans cells and dermal dendritic cells (dDCs)) [38,39,40] which migrate to the local draining lymph nodes (dLNs), where they activate allergen-specific naïve T cells through the following: (i) the presentation of allergen adduct-containing major histocompatibility complexes (MHC I and II) recognized by T cell receptors (TCRs) on the T cells [41,42]; (ii) the expression of the co-stimulatory molecules CD80 and CD86 engaging the CD28 co-receptor on the T cells [43]; and (iii) the release of polarizing cytokines that drive T cell subset differentiation [44,45]. After several days of proliferation (5–7 days in mice and 10–15 days in humans), a high number of allergen-specific effector T cells, with the ability to produce effector cytokines and move into the circulation, are generated in the dLNs [1,46,47]. A large fraction of these allergen-specific effector T cells express skin-homing molecules, including C-X-C motif receptor (CXCR) 3, which enable them to migrate to the affected skin site following a chemokine gradient, including the C-X-C motif ligands (CXCL) 9, 10, and 11 [48,49,50,51,52]. In a highly targeted manner, the T cells then clear keratinocytes presenting allergen adduct-expressing MHC molecules, whereafter most of the effector T cells undergo apoptosis. However, subsets of allergen-specific T cells develop into memory T cells that persist in the skin, circulation, and secondary lymphoid organs [1] (Figure 1).

## 3. The Role of Neutrophils in the Sensitization Phase

Neutrophils have been described to play an important role during sensitization and challenge to contact allergens. During sensitization, Weber et al. (2015) [21] showed that neutrophils were required for contact allergen-induced release of cytokines, the migration of DCs to the dLNs, and the priming of allergen-specific T cells. The early recruitment of neutrophils to the skin following initial allergen exposure was essential for directing the subsequent infiltration of effector CD8^+^ T cells to the skin [21]. The central role of neutrophils in the sensitization phase is supported by the observation that myeloperoxidase (MPO), a neutrophil-specific mediator of cell damage, is required for the release of IL-1β during sensitization [53]. Neutrophils direct T cells into the skin following exposure to DNFB through expression of FasL and perforin [23] and the-release of the T cell recruiting chemokine interferon gamma (IFN-γ)-induced protein 10 (CXCL10/IP-10) [54]. The rapid entry of neutrophils 3–6 h after exposure to the contact allergen during the sensitization phase depends on IL-1 receptor (IL-1R)-mediated activation of mast cells, dDCs [21], and IL-17-producing γδ T cells in the dermis [55]. Accordingly, IL-1R^−^/^−^ mice exhibit reduced neutrophil and CD8^+^ T cell infiltration in skin exposed to DNFB [56]. Collectively, these findings suggests that infiltration of neutrophils in skin exposed to contact allergens is required to enable subsequent entry of allergen-specific effector T cells during the sensitization phase (Figure 1).

Another important aspect of neutrophil involvement in ACD is the feature of pruritus that often follows after exposure to contact allergens [1]. Transient receptor potential (TRP) ion channels expressed by sensory neurons in the skin enhance ACD responses by stimulating itch and scratching behavior following exposure to contact allergens [57]. The mechanical stress from scratching triggers elevated levels of cytokines (IL-1β, IL-6, TNF-α) and chemokines (CXCL1, CXCL2, CXCL5), leading to increased neutrophil infiltration in the skin [58]. In addition, neutrophil-derived leukotriene B4 (LTB4), released in response to mechanical skin stress (e.g., scratching or tape stripping), further promotes neutrophil recruitment [59]. Notably, both scratching behavior and LTB4-driven neutrophil accumulation were found to be essential for ACD induction [59]. Neutrophil-induced oxidative stress significantly enhances local release of the pruritic mediator IL-33 in the skin, suggesting that neutrophils may also act as a trigger or amplifier of scratching behavior following exposure to contact allergens [60] (Figure 1).

## 4. The Function of Memory CD8^+^ T Cells and Neutrophils During the Challenge Phase

**Figure 2:**
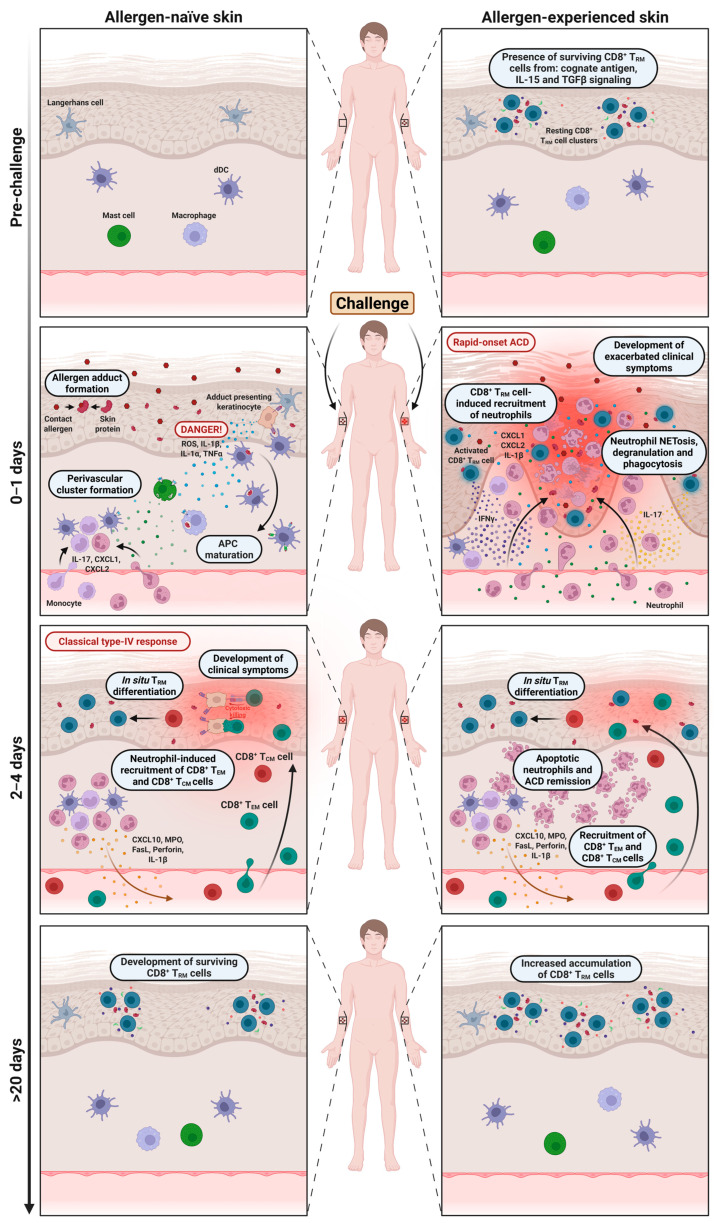
The challenge phase in allergen-naïve versus allergen-experienced skin. In contrast to allergen-naïve skin, allergen-experienced skin harbors allergen-specific CD8^+^ tissue-resident memory T cells (T_RM_), which determines the kinetics and severity of the inflammatory response following allergen re-exposure. Upon re-exposure to the contact allergen, CD8^+^ T_RM_ cells in allergen-experienced skin are rapidly activated and within 0–1 days release interferon-γ (IFN-γ) and interleukin-17 (IL-17) that induce the production of chemokines such as C-X-C motif ligand 1/2 (CXCL1/2) which drive massive infiltration of neutrophils and tissue damage. In contrast, naïve skin responds more slowly with allergen adduct formation, danger signaling, and immune cell clustering, as seen during the sensitization phase. By 2–4 days, inflammation in allergen-experienced skin resolves as neutrophils die, while in allergen-naïve skin, CD8^+^ effector memory (T_EM_) T cells infiltrate the skin and produce a classical delayed-type ACD reaction. After >20 days, both skin sites accumulate allergen-specific CD8^+^ T_RM_ cells, enabling rapid-onset ACD upon re-challenge at any of the skin sites. Other abbreviations: ROS, reactive oxygen species; TNF-α, tumor necrosis factor-α; MPO, myeloperoxidase; FasL, Fas ligand.

Whereas the sensitization phase is typically asymptomatic, the challenge phase elicits the classical clinical symptoms of ACD. Importantly, the dynamics of this phase differ depending on whether the skin has previously been exposed to the contact allergen (Figure 2 and Table 1). Memory T cells are central to the response in both contexts; however, allergen-experienced skin uniquely harbors allergen-specific CD8^+^ T_RM_ cells, whereas allergen-naïve skin does not.

In 1963, Arnason and Waksman were the first to report the existence of local memory to contact allergens at skin sites previously exposed to the given contact allergen, a phenomenon they termed “retest reactions” [61]. This sparked a debate about whether local immunological memory may develop in the skin [62,63]. Yet the critical role of CD8^+^ T_RM_ cells in local immunological memory was not demonstrated until much later. A pivotal study by Gebhardt et al. (2009) identified skin CD8^+^ T_RM_ cells in herpes simplex virus-infected skin that facilitated local protection against reinfections [64]. Later, Gaide et al. (2015) showed that CD8^+^ T_RM_ cells developed and persisted locally in skin exposed to the contact allergen dinitrofluorobenzene (DNFB) [65]. By use of a parabiotic mouse model, they further showed that the presence of skin-resident CD8^+^ T_RM_ cells correlated with an enhanced, rapid challenge response that peaked approximately 24 h after exposure to the allergen. Accordingly, Gadsbøll et al. (2020) showed that the number of epidermal CD8^+^ T_RM_ cells increases with repeated exposures to DNFB and correlates with the magnitude of the challenge response [66].

During the first 24 h after exposure of allergen-naïve skin to the contact allergen, only minimal inflammation is observed. Local innate immune cells recognize the allergen and initiate the processes described in Section 3. Compared to sensitization, the onset of inflammation in allergen-naïve skin is somewhat accelerated due to the presence of allergen-specific CD8^+^ central memory (T_CM_) cells in the draining lymph nodes (dLNs), which proliferate rapidly upon arrival of mature APCs, and the presence of allergen-specific CD8^+^ effector memory (T_EM_) cells in the circulation [65,67,68]. These memory populations infiltrate the challenged skin within 2–4 days, thereby facilitating the classical type IV hypersensitivity response that typically peaks 48–72 h after challenge at an allergen-naïve skin site [4,13,69]. Following resolution, CD8^+^ T_RM_ cells are established at the site, rendering the previously allergen-naïve skin allergen-experienced.

In contrast to allergen-naïve skin, re-exposure of allergen-experienced skin to the sensitizing allergen, as typically occurs in daily life and in some occupational settings, triggers a more rapid and pronounced inflammatory response. In this condition, the epidermis already harbors allergen-specific CD8^+^ T_RM_ cells strategically positioned to respond immediately once the allergen penetrates the skin barrier [7,13,66,70]. Within a few hours, the T_RM_ cells initiate rapid-onset ACD by releasing pro-inflammatory cytokines, most prominently IFN-γ and IL-17 [7,71]. Mechanistically, IFN-γ upregulates MHC I/II and Fas expression on keratinocytes [72,73] and induces secretion of IL-1β, TNF-α, and ROS [71,74], while IL-17, in synergy with IFN-γ, serves as a key upstream driver of neutrophil recruitment [71,75,76]. The critical role of IL-17-producing T cells during the challenge phase has been demonstrated in TCR δ^−^/^−^ and IL-17R^−^/^−^ mice, both of which fail to recruit neutrophils upon DNFB challenge, in contrast to IFN-γR^−^/^−^ mice [55,71]. Accordingly, IL-17 expression and neutrophil accumulation are prominent features of the challenge reaction in nickel-experienced skin of patients allergic to nickel [7,16].

Consistent with these findings, activated T_RM_ cells induce rapid production of neutrophil-recruiting chemokines, including CXCL1 and CXCL2, that drive massive neutrophil infiltration and subsequent tissue damage [13,14,15,16,25,75,76,77]. Both chemokines act through CXCR2, as evidenced by markedly reduced neutrophil infiltration in CXCR2^−^/^−^ mice [22] and following pharmacological inhibition with the CXCR2 antagonist Reparixin [13]. The essential contribution of neutrophils in rapid-onset ACD has further been demonstrated by antibody-mediated depletion of neutrophils [13]. Moreover, neutrophil extracellular trap (NETosis) formation in response to DNFB challenge has recently been identified as an additional effector mechanism amplifying tissue inflammation [78], highlighting the pathological function of neutrophils in the challenge phase.

Whereas the classical type IV response in allergen-naïve skin peaks 48–72 h after challenge, inflammation in allergen-experienced skin has already started to resolve by this time, as skin-infiltrating neutrophils quickly undergo cell death [13]. In parallel, CD8^+^ T_EM_ cells start to infiltrate the site, further promoting the expansion of allergen-specific epidermal CD8^+^ T_RM_ cells that reinforce long-term reactivity to the contact allergen [13,66].

**Table 1:** Differences between allergen-naïve and -experienced skin upon challenge.

Feature	Challenge with Contact Allergen on:		
	Allergen-Naïve Skin	Allergen-Experienced Skin	Refs.
Resulting skin reaction	Classical delayed-type (type IV) hypersensitivity reaction. Typically seen when patch testing patients.	Rapid-onset and exacerbated reaction. Typically seen in daily life situations where patients are re-exposed at the same skin site repeatedly.	[5,6,7,13,15]
Presence of allergen-specific T_RM_ cells	Absent.	CD8^+^ T_RM_ cells enriched in epidermis.	[7,13,15]
Kinetics	Delayed-type response (peaks ~48–72 h post challenge).	Rapid-onset response (<24 h post challenge).	[5,6,7,13,15]
Memory T cell subsets involved in inflammatory response	Circulating and effector memory T cell subsets (T_CM_ and T_EM_ subsets).	CD8^+^ T_RM_ cells (predominantly Tc1 and Tc17 subsets).	[7,13,15,79]
Cytokines/molecules involved	IL-1β, IL-12, and CXCL10 (late induction); IFN-γ, IL-17, and TNF-α (produced after T cell recruitment).	IL-1β, IFN-γ, IL-17, TNF-α, and granzyme B, perforin (produced locally and early); CXCL1 and CXCL2 (recruitment of neutrophils).	[7,13,15,22,36,44,49,50,80]

## 5. Mediators of Long-Term Persistence of Skin-Resident CD8^+^ T_RM_ Cells

A key feature of T_RM_ cells is their ability to adapt to and persist in the dynamic and energy-constrained environment found in peripheral tissues. In the epidermis, allergen-specific CD8^+^ T_RM_ cells compete with each other and with other T cell subsets for metabolites, survival signals, and the limited anatomical space [66,81,82,83]. IL-15 and transforming growth factor (TGF)-β are essential for the development of CD8^+^ T_RM_ cells [83,84]. IL-15 is constitutively produced by keratinocytes in the hair follicles and appears to play a crucial role in supporting CD8^+^ T_RM_ cell survival in the epidermis [82]. Accordingly, IL-15 signaling has been implicated in CD8^+^ T_RM_ cell-mediated pathology in several autoimmune skin diseases [85], and blockade of the IL-15 receptor has been proposed for treatment of vitiligo [86] and alopecia areata [87], two skin conditions that are driven by pathologic CD8^+^ T_RM_ cells. More recently, TGF-β-induced downregulation of sphingosine-1-phosphate receptor 5 (S1PR5) expression in CD8^+^ T_RM_ cells has been identified as a key mechanism promoting the long-term persistence of CD8^+^ T_RM_ cells in the skin [88]. In contrast, IL-4 impairs CD8^+^ T_RM_ development by inhibiting TGF-β-induced expression of CD103 [89]. Together, these data support that different cytokines, transcription factors, and surface receptors involved in CD8^+^ T_RM_ cell development and persistence could be targets in future treatment of rapid-onset ACD and other CD8^+^ T_RM_ cell-driven skin diseases.

The role of continuous antigen presentation in the long-term persistence of skin CD8^+^ T_RM_ cells has been a subject of debate [31,90,91,92,93]. Most current evidence suggests that while local inflammatory cues can drive effector CD8^+^ T cells to migrate into the skin and transiently adopt a T_RM_ phenotype in the absence of cognate antigen, their long-term persistence depends on the continuous presence of allergen adducts and TCR signaling [31,91,92,93]. It has further been shown that CD8^+^ T_RM_ cells tend to localize near epidermal allergen adducts and that the number and diversity of T cells decline in parallel with decreasing levels of allergen adducts in the epidermis over time [31,70]. Importantly, not all contact allergens are capable of supporting long-term T_RM_ survival [31], which aligns with findings showing that only some contact allergens can propagate the exacerbated neutrophil-mediated reactions observed in rapid-onset ACD [15]. This suggests that the half-life of allergen adducts in the skin influences the persistence of CD8^+^ T_RM_ cells.

## 6. Potential Therapeutic Targets Against Rapid-Onset ACD

Currently, there is no curative treatment for individuals who have developed contact allergy. Management of ACD primarily focuses on allergen avoidance strategies and topical or systemic corticosteroids to alleviate acute flare-ups [1,94]. Studies in mice have demonstrated that corticosteroid treatments have limited or negligible effects on the development and survival of allergen-specific CD8^+^ T_RM_ cells [95]. There is no current treatment addressing the underlying specific immune response behind rapid-onset nor delayed-type ACD. The recent advances in understanding the immunopathology of rapid-onset ACD have uncovered several promising therapeutic targets aimed at mitigating the immune responses driven by allergen-specific CD8^+^ T_RM_ cells and their recruitment of neutrophils. Here, we wish to review potential therapeutic strategies with a particular focus on targeting rapid-onset ACD. While the following therapeutic approaches are mechanistically intriguing, their clinical applicability in treating ACD remains largely uncertain. Each of the proposed strategies presents specific limitations and potential side effects. A summary of these potential therapeutic strategies and their limitations is provided in Table 2.

### 6.1. Targeting the IL-1R-CXCR2 Neutrophil Axis

IL-1β acts as a local danger signal during sensitization, and IL-1α drives dermal macrophage production of CXCL2, promoting perivascular immune-cell cluster formation and augmenting CD8^+^ T cell activation and expansion in the skin [33,96]. Accordingly, preclinical mouse studies indicate that blockade of IL-1R [33,97] or CXCR2 [13,22] reduces neutrophil recruitment and downstream T cell-dependent pathology and therefore represents an attractive therapeutic strategy.

However, translation of these therapies to humans is at an early stage. An interventional trial testing IL-1 blockade (Anakinra) in ACD is registered (NCT05498467), and CXCR2 antagonists have been evaluated in several clinical trials against neutrophil-driven implications (for example COPD/bronchiectasis and oncology studies) [98,99,100,101,102], but, to our knowledge, not yet in randomized trials for ACD. Importantly, CXCR2 inhibitors produce reversible reductions in the number of circulating and tissue neutrophils within 24 h in humans [99], demonstrating the pharmacodynamic feasibility of targeting this pathway; however, the clinical efficacy and safety of their use in ACD are still not clear [98,99,100,101,102]. A practical limitation for IL-1R–CXCR2 targeting in rapid-onset ACD is the need for near-immediate administration following allergen re-exposure to prevent neutrophil extravasation. Thus, timing of treatment may limit practical applicability and argues for exploring short-acting topical/locally delivered formulations or rapid-access treatment pathways in future trials for ACD. Moreover, as neutrophils are critical for first-line host defense and wound healing, therapeutic inhibition of neutrophil recruitment may increase susceptibility to infections and impair tissue repair, as reported in clinical studies of systemic CXCR2 blockade [101,102].

### 6.2. Neutralization Inflammatory Cytokines (IL-17/IFN-γ)

CD8^+^ T_RM_ cells contribute to early neutrophil recruitment in rapid-onset ACD by secreting IL-17 and IFN-γ, which in turn induce the release of CXCL1 and CXCL2 [7,13,16,55,71]. In preclinical models, blockade of IL-17 or IFN-γ signaling has demonstrated efficacy, and several monoclonal antibodies targeting these cytokines, such as Secukinumab (anti-IL-17) [103] and Emapalumab (anti-IFN-γ), exist [104]. Furthermore, neutralization of IL-17 has shown strong efficacy in the treatment of psoriasis [105,106,107]. Given the proposed role of IL-17 in neutrophil recruitment during rapid-onset ACD [7,13,16], it indicates that IL-17 blockade may also be beneficial for ACD patients.

Yet a trial of Secukinumab in nickel-allergic patients demonstrated little-to-no clinical effect after patch testing [108], suggesting that IL-17 is not a major driver of the classical type IV response to nickel, which of course does not rule out that IL-17 plays an important role in rapid-onset ACD. However, a key concern especially with systemic IL-17 blockade is safety as multiple clinical trials (e.g., in psoriasis) showed a significantly increased risk of mucocutaneous candidiasis (oral, cutaneous, and oropharyngeal) with IL-17 inhibitors compared with placebo or non-IL-17 biologics [109,110]. Again, short-acting topical/locally delivered formulations would be preferrable.

### 6.3. JAK Inhibitors

IL-15, IL-17, and IFN-γ all signal through pathways involving Janus kinases (JAKs). JAK inhibitors, such as Tofacitinib and Ruxolitinib, have shown promise in the treatment of CD8^+^ T_RM_-driven inflammatory skin disorders, including vitiligo [111] and alopecia areata [112,113], and both systemic agents (Abrocitinib, Upadacitinib, Baricitinib) and topical Ruxolitinib cream are approved for the treatment of atopic dermatitis [111,114]. Their ability to suppress cytokine signaling downstream of IFN-γ, IL-4, and IL-17 makes JAK inhibitors attractive candidates for ACD, especially when applied topically, as systemic JAK inhibition carries recognized risks, including thromboembolic events, cytopenia, and serious infections [115,116]. Topical formulations may reduce some of these risks, but their efficacy and safety in ACD remain largely unexplored. Considering the efficacy shown in other local and CD8^+^ T_RM_-driven skin diseases, topically applied JAK inhibitors represent a promising treatment strategy for rapid-onset ACD.

### 6.4. Modulation of CD8^+^ T_RM_ Cell Survival and Function

The persistence of CD8^+^ T_RM_ cells in the epidermis depends on IL-15 and TGF-β signaling [83]. Local inhibition of these pathways, for example via monoclonal antibodies targeting IL-15/CD122 signaling [117] or TGF-β [118], may effectively reduce T_RM_ cell survival. Additionally, recombinant cytokines such as IL-4, which has been shown to counteract CD8^+^ T_RM_ cell survival [89], could offer an alternative strategy to inhibit T_RM_ cell survival. IL-4 downregulates the expression of CD103, CD49a, and TGF-β receptor II on CD8^+^ T cells, thus impairing T_RM_ formation in skin, and in atopic dermatitis lesions, IL-4 expression correlates with reduced T_RM_ formation [89].

Despite encouraging preclinical data, no clinical trials have yet evaluated IL-15, TGF-β, or IL-4-based interventions in ACD. Translating these approaches into ACD is challenging because both IL-15 and TGF-β are pleiotropic cytokines with essential roles in immune homeostasis and tissue repair. Blockade of IL-15 signaling risks impairing antiviral and antitumor immunity, while TGF-β inhibition may cause widespread immune dysregulation and promote autoimmune or inflammatory pathology. Recombinant IL-4, while potentially useful for modulating T_RM_ cell survival, could exacerbate type 2 inflammation and worsen conditions such as atopic dermatitis or asthma. Yet, to our knowledge, specific adverse effects in humans with ACD or other immune skin defects have not been studied. Thus, while modulation of T_RM_ survival through IL-15, TGF-β, or IL-4 pathways represents a conceptual curative strategy for rapid-onset ACD, its clinical feasibility and safety remain uncertain and require cautious investigation in human trials.

### 6.5. Stimulation of Inhibitory Checkpoint Receptor Signaling

PD-1 is an inhibitory receptor expressed on T cells that, upon engagement with its ligands PD-L1 and PD-L2, downregulates T cell activation and promotes immune tolerance [119]. Experimental models have shown promise in enhancing the expression or signaling of inhibitory checkpoint receptors, e.g., by treatment with PD-1 agonists [120]. This may raise the activation threshold of CD8^+^ T_RM_ cells, thereby suppressing T_RM_-induced inflammation and preventing the development of dermatitis [70]. Similarly, cytotoxic T-lymphocyte-associated protein 4 (CTLA-4), another inhibitory receptor, has been shown to suppress pathogenic T cells in an atopic dermatitis mouse model [121]. However, several limitations and potential risks exist with this approach. Systemic administration of checkpoint agonists may cause profound immunosuppression, increasing the susceptibility to infections and malignancies. However, new drugs that can be topically applied and enhance inhibitory checkpoint receptor signaling might hold promise in the treatment of ACD.

### 6.6. Targeting Metabolic Pathways

Metabolic reprogramming via the AhR and mTOR signaling pathways has regulated CD8^+^ T_RM_ cell development, function, and survival in preclinical mouse studies [66,122,123,124]. In addition, targeting AhR with modulators such as Tapinarof has been shown to downregulate IL-17 responses in both mice and humans [125]. Thus, modulating these pathways may offer an effective strategy to alter both CD8^+^ T_RM_ cell activation and persistence in the skin. Tapinarof has been proven effective in clinical trials for psoriasis [126,127,128] and atopic dermatitis [129,130], with adverse events reported including folliculitis, nasopharyngitis, upper respiratory tract infection, headache, and contact dermatitis [127]. mTOR inhibitors like rapamycin modulate T cell responses, alleviating autoimmune diseases in mice [131,132] and humans [133]. Systemic use of rapamycin has several well-documented adverse effects including enhanced risk of infections, dyslipidemia, mouth ulcers, and occasionally renal or metabolic abnormalities [133,134], so careful monitoring is required in clinical use. To our knowledge, AhR and mTOR pathway modulation in ACD has not been studied. However, topical targeting of metabolic pathways may be an avenue for ACD therapy, but further studies are needed to establish its safety and efficacy in humans.

### 6.7. Anti-Pruritic Strategies

As scratching exacerbates skin inflammation and allergen penetration, targeting itch-related pathways, such as TRPA1, IL-33, LTB4, or histamine receptors, could indirectly reduce the severity of ACD by preventing mechanical skin barrier disruption and neutrophil recruitment [57,58,59,60].

While leukotriene receptor antagonists (LTRAs) are clinically used for other pruritic or inflammatory skin conditions [135,136], no trials have specifically tested these in ACD. Similarly, TRPA1 antagonists (Montelukast) have shown efficacy in reducing itch/pain [137], but no dermatology-specific clinical trials have been completed to our knowledge. Biologics targeting IL-33 (e.g., Etokimab, Tozorakimab) have entered testing in atopic dermatitis, with early proof-of-concept data suggesting reduced disease activity and pruritus, including a significant reduction in neutrophil skin infiltrates [138]. Subsequent phase 2 trials have reported mixed efficacy [139]. In contrast, H_1_-antihistamines remain the primary agent of symptomatic anti-pruritic therapy in dermatology, particularly for urticaria and other histamine-dependent pruritic dermatoses [140], although their efficacy in non-histaminergic itch seem limited, suggesting no or minor efficacy in ACD.

Potential adverse effects also need to be considered when targeting itch pathways. H_1_-antihistamines are generally well tolerated, but drowsiness, fatigue, and impaired concentration and memory are reported due to their ability to cross the blood–brain barrier. Yet second-generation antihistamines, including loratadine, seem less likely to cause sedation, but may still have mild central nervous system effects [141,142]. IL-33 antagonists have shown a favorable safety profile in clinical trials for atopic dermatitis, although a single adverse event of venous thrombosis was reported [139]. However, larger studies are needed to fully assess their long-term safety. TRPA1 antagonists have demonstrated safety in early-phase clinical trials, with no significant adverse events reported [143]. Nonetheless, due to the broad expression of TRPA1 in various tissues, potential off-target effects warrant further investigation.

### 6.8. Redox Modulation and Allergen Metabolism

Variations in allergen persistence seem to influence the ability of CD8^+^ T_RM_ cells to survive [31,69,144]. The Nrf2 transcription factor pathway plays a key role in the detection and detoxification of cytosolic allergen adducts [28,145]. Thus, enhancing Keap1-Nrf2 signaling to regulate the skin’s redox balance and facilitate allergen clearance could represent a strategy to reduce allergen adducts and ROS-driven skin inflammation. In this context, antioxidants, including sulforaphane and curcumin, are potent activators of the Nrf2 pathway that have been shown to modulate oxidative stress and inflammatory responses [146]. While preclinical studies support these effects, clinical trials in ACD are lacking, and systemic administration of Nrf2 activators may carry off-target effects, again highlighting the need for further research to establish efficacy for ACD and safety in humans.

**Table 2:** Potential therapeutic strategies for rapid-onset ACD.

Therapeutic Strategy	Mechanism	Limitations	Reported/Potential Side Effects	Refs.
Anti-IL-1 and anti-IL-1R blockade (e.g., Anakinra, CXCR2 inhibitors)	Reduces neutrophil recruitment and downstream CD8^+^ T_RM_ activation	Requires near-immediate administration after exposure; limited data in ACD	Neutropenia, increased infection risk; may transiently impair host defense	[13,22,33,96,97,98,99,100,101,102]
Cytokine neutralization (anti-IL-17/anti-IFN-γ) (e.g., Secukinumab, Emapalumab)	Blocks CD8^+^ T_RM_-induced inflammation and downstream release of neutrophil-recruiting chemokines	Secukinumab showed limited efficacy in a nickel allergy trial; unknown efficacy against rapid-onset ACD	IL-17 inhibitors: increased risk of mucocutaneous candidiasis; IFN-γ blockade: increased infection risk	[7,71,103,104,105,106,107,108,109,110]
JAK inhibitors (e.g., Tofacitinib, Ruxolitinib, Abrocitinib, Upadacitinib, Baricitinib)	Broad inhibition of cytokine signaling (IL-15, IL-17, IFN-γ, IL-4) and inflammation	Systemic use limited by safety concerns; efficacy in ACD unknown; topical efficacy in ACD is unexplored	Systemic: thromboembolism, cytopenia, and serious infections; topical: local irritation and unknown long-term safety	[111,112,113,114,115,116]
CD8^+^ T_RM_ modulation (e.g., anti–IL-15, anti–TGF-β, IL-4 administration)	Reduces CD8^+^ T_RM_ survival and persistence	No ACD trials; IL-15 and TGF-β are pleiotropic; IL-4 may worsen or cause development of type 2 autoimmunity.	IL-15 blockade may impair antiviral/antitumor immunity; TGF-β blockade increases risk of autoimmunity/inflammation; IL-4 may exacerbate AD/asthma	[83,89,117,118,147]
Checkpoint receptor agonists (e.g., PD-1, CTLA-4 agonists)	Inhibit T cell activation and dampen T_RM_-driven inflammation	No clinical trials in skin disease; systemic immunosuppression risk; topical formulations not available	Increased infection and malignancy risk with systemic administration	[70,119,120,121]
Metabolic pathway modulation (AhR modulators e.g., Tapinarof; mTOR inhibitors, e.g., rapamycin)	Alters T_RM_ activation/inflammation and survival; Tapinarof is shown to reduce IL-17 expression	No studies in ACD; paradoxical of contact dermatitis with Tapinarof; systemic rapamycin toxicity	Tapinarof: folliculitis, nasopharyngitis, and contact dermatitis; rapamycin: infections, dyslipidemia, mouth ulcers, and renal/metabolic toxicity	[66,122,123,124,125,126,127,128,129,130,131,132,133,134,148]
Anti-pruritic strategies (e.g., H_1_-antihistamines, TRPA1 antagonists, LTRAs, IL-33 blockade)	Reduce scratching-induced barrier damage and neutrophil infiltration	Limited efficacy in non-histaminergic itch; mixed efficacy in AD (IL-33); no ACD trials	H_1_-antihistamines: sedation, fatigue, and cognitive impairment; IL-33 blockade: rare thrombosis; TRPA1 antagonists: possible off-target effects; LTRAs: safe in asthma, but untested in ACD	[57,58,59,60,135,136,137,138,139,140,141,142,143]
Redox modulation/allergen metabolism (e.g., Nrf2 activators: sulforaphane, curcumin)	Enhances detoxification of allergen adducts and reduces oxidative stress	Only preclinical data; systemic administration may cause off-target effects	Safety in ACD not established	[28,31,69,144,145,146]

## 7. Conclusions and Perspectives

Importantly, by distinguishing rapid-onset ACD from the classical delayed-type reaction, this review highlights a clinically distinct manifestation that is highly relevant for patients who experience sudden flare-ups in daily life. Recognizing this difference is crucial for accurate diagnosis and the development of therapeutic strategies tailored to rapid-onset ACD, which is currently underrepresented in the literature. Collectively, this review extends current knowledge by providing a comparative perspective on rapid-onset and delayed-type ACD. Whereas delayed-type ACD is dominated by T cell responses peaking after 48–72 h, rapid-onset ACD involves immediate CD8^+^ T_RM_ cell activation and neutrophil-driven inflammation, leading to acute clinical flare-ups. This contrast not only clarifies discrepancies between experimental models, patch testing, and daily patient experiences, but also carries translational implications for therapy, emphasizing the need to specifically target mechanisms unique to rapid-onset disease. The recognition of CD8^+^ T_RM_ cells and neutrophils as central players in rapid-onset ACD has opened new avenues for therapeutic interventions regarding ACD. Approaches aimed at modulating T_RM_ cell survival, function, and activation thresholds including targeting central cytokine pathways, altering the metabolic and transcriptional environment, and disrupting neutrophil recruitment are proposed in this review.

While the therapeutic strategies discussed here highlight the potential for mechanism-based treatment of ACD, their translation to clinical practice faces significant challenges. Most remain untested in ACD patients, and safety profiles may be unfavorable for a condition often managed effectively by allergen avoidance. The accelerated kinetics of rapid-onset ACD further complicates intervention, as patients are frequently unaware of recent allergen exposure at the time symptoms develop. Therefore, avoidance strategies will likely continue to be the main strategy for contact allergic patients. Nonetheless, for individuals at a high risk of repeated exposure, such as those in certain occupations, a novel, fast-acting, local/topical, and easy-to-administer therapy capable of halting or attenuating rapid-onset ACD could be highly beneficial. Such interventions could reduce acute inflammation, prevent disruption of skin barrier function, and limit the downstream recruitment of pathogenic immune cells, thereby preserving work ability and lowering the current cost for society. Future studies should focus on evaluating these strategies in well-designed preclinical and clinical trials, with careful attention paid to their safety and feasibility and the unique kinetics of rapid-onset versus delayed-type ACD.

## Data Availability

No new data were created or analyzed in this study. Data sharing is not applicable to this article.

## Abbreviations

The following abbreviations are used in this manuscript:

ACD	Allergic contact dermatitis
AhR	Aryl hydrocarbon receptor
APC	Antigen-presenting cell
CCR7	C-C chemokine receptor type 7
CXCL	C-X-C motif chemokine ligand
CXCR	(C-X-C motif) receptor
dDC	Dermal dendritic cell
dLN	Draining lymph node
DNFB	Dinitrofluorobenzene
FasL	Fas ligand
IFN-γ	Interferon-γ
IL	Interleukin
IL-1R	Interleukin-1 receptor
JAK	Janus kinase
LTB4	Leukotriene B4
LTRA MHC	Leukotriene receptor antagonist Major histocompatibility complex
MPO	Myeloperoxidase
mTOR	Mechanistic target of rapamycin
NETosis	Neutrophil extracellular trap formation
Nrf2	Nuclear factor erythroid 2–related factor 2
PD-1	Programmed cell death protein 1
ROS	Reactive oxygen species
TCR	T cell receptor
T_CM_	Central memory T cell
T_EM_	Effector memory T cell
TGF-β	Transforming growth factor-β
TNF-α	Tumor necrosis factor-α
T_RM_	Tissue-resident memory T cell
TRP	Transient receptor potential

## References

[1] Scheinman P.L., Vocanson M., Thyssen J.P., Johansen J.D., Nixon R.L., Dear K., Botto N.C., Morot J., Goldminz A.M. (2021). Contact Dermatitis. Nat. Rev. Dis. Prim..

[2] Svensson A., Ofenloch R.F., Bruze M., Naldi L., Cazzaniga S., Elsner P., Goncalo M., Schuttelaar M.-L.A., Diepgen T.L. (2018). Prevalence of Skin Disease in a Population-Based Sample of Adults from Five European Countries. Br. J. Dermatol..

[3] Tramontana M., Hansel K., Bianchi L., Sensini C., Malatesta N., Stingeni L. (2023). Advancing the Understanding of Allergic Contact Dermatitis: From Pathophysiology to Novel Therapeutic Approaches. Front. Med..

[4] Johansen J.D., Aalto-Korte K., Agner T., Andersen K.E., Bircher A., Bruze M., Cannavõ A., Giménez-Arnau A., Gonçalo M., Goossens A. (2015). European Society of Contact Dermatitis Guideline for Diagnostic Patch Testing—Recommendations on Best Practice. Contact Dermat..

[5] Ahlström M.G., Menné T., Thyssen J.P., Johansen J.D. (2017). Nickel Allergy in a Danish Population 25 Years after the First Nickel Regulation. Contact Dermat..

[6] Hindsén M., Bruze M., Christensen O.B. (1997). The Significance of Previous Allergic Contact Dermatitis for Elicitation of Delayed Hypersensitivity to Nickel. Contact Dermat..

[7] Schmidt J.D., Ahlström M.G., Johansen J.D., Dyring-Andersen B., Agerbeck C., Nielsen M.M., Poulsen S.S., Woetmann A., Ødum N., Thomsen A.R. (2017). Rapid Allergen-induced Interleukin-17 and Interferon-γ Secretion by Skin-resident Memory CD8 + T Cells. Contact Dermat..

[8] Johansen J.D., Bonefeld C.M., Schwensen J.F.B., Thyssen J.P., Uter W. (2022). Novel Insights into Contact Dermatitis. J. Allergy Clin. Immunol..

[9] Frosch P.J., Kügler K. (2011). Occupational Contact Dermatitis. Contact Dermatitis.

[10] Pesonen M., Jolanki R., Larese Filon F., Wilkinson M., Kręcisz B., Kieć-Świerczyńska M., Bauer A., Mahler V., John S.M., Schnuch A. (2015). Patch Test Results of the European Baseline Series among Patients with Occupational Contact Dermatitis across Europe—Analyses of the European Surveillance System on Contact Allergy Network, 2002–2010. Contact Dermat..

[11] Zuberbier T., Lötvall J., Simoens S., Subramanian S.V., Church M.K. (2014). Economic Burden of Inadequate Management of Allergic Diseases in the European Union: A GA(2)LEN Review. Allergy.

[12] Saetterstrøm B., Olsen J., Johansen J.D. (2014). Cost-of-Illness of Patients with Contact Dermatitis in Denmark. Contact Dermat..

[13] Funch A.B., Mraz V., Gadsbøll A.Ø., Jee M.H., Weber J.F., Ødum N., Woetmann A., Johansen J.D., Geisler C., Bonefeld C.M. (2022). CD8+ Tissue-Resident Memory T Cells Recruit Neutrophils That Are Essential for Flare-Ups in Contact Dermatitis. Allergy.

[14] Mraz V., Funch A.B., Jee M.H., Gadsbøll A.-S.Ø., Weber J.F., Yeung K., Lohmann R.K.D., Hawkes A., Ødum N., Woetmann A. (2023). CD100 Boosts the Inflammatory Response in the Challenge Phase of Allergic Contact Dermatitis in Mice. Contact Dermat..

[15] Funch A.B., Weber J.F., Lohmann R.K.D., Mraz V., Yeung K., Jee M.H., Ødum N., Woetmann A., Johansen J.D., Geisler C. (2023). CD4+ T Cells Inhibit the Generation of CD8+ Epidermal-Resident Memory T Cells Directed against Clinically Relevant Contact Allergens. Contact Dermat..

[16] Funch A.B., Ahlström M.G., Johansen J.D., Geisler C., Bonefeld C.M. (2024). Neutrophil Infiltration in Allergic Contact Dermatitis to Nickel. Br. J. Dermatol..

[17] Goebeler M., Trautmann A., Voss A., Bröcker E.B., Toksoy A., Gillitzer R. (2001). Differential and Sequential Expression of Multiple Chemokines during Elicitation of Allergic Contact Hypersensitivity. Am. J. Pathol..

[18] Larsen J.M., Bonefeld C.M., Poulsen S.S., Geisler C., Skov L. (2009). IL-23 and T(H)17-Mediated Inflammation in Human Allergic Contact Dermatitis. J. Allergy Clin. Immunol..

[19] Burn G.L., Foti A., Marsman G., Patel D.F., Zychlinsky A. (2021). The Neutrophil. Immunity.

[20] Kolaczkowska E., Kubes P. (2013). Neutrophil Recruitment and Function in Health and Inflammation. Nat. Rev. Immunol..

[21] Weber F.C., Németh T., Csepregi J.Z., Dudeck A., Roers A., Ozsvári B., Oswald E., Puskás L.G., Jakob T., Mócsai A. (2015). Neutrophils Are Required for Both the Sensitization and Elicitation Phase of Contact Hypersensitivity. J. Exp. Med..

[22] Cattani F., Gallese A., Mosca M., Buanne P., Biordi L., Francavilla S., Coletti G., Pellegrini L., Melillo G., Bertini R. (2006). The Role of CXCR2 Activity in the Contact Hypersensitivity Response in Mice. Eur. Cytokine Netw..

[23] Kish D.D., Gorbachev A.V., Parameswaran N., Gupta N., Fairchild R.L. (2012). Neutrophil Expression of Fas Ligand and Perforin Directs Effector CD8 T Cell Infiltration into Antigen-Challenged Skin. J. Immunol..

[24] Kish D.D., Min S., Dvorina N., Baldwin W.M., Stohlman S.A., Fairchild R.L. (2019). Neutrophil Cathepsin G Regulates Dendritic Cell Production of IL-12 during Development of CD4 T Cell Responses to Antigens in the Skin. J. Immunol..

[25] Dilulio N.A., Engeman T., Armstrong D., Tannenbaum C., Hamilton T.A., Fairchild R.L. (1999). Groα-Mediated Recruitment of Neutrophils Is Required for Elicitation of Contact Hypersensitivity. Eur. J. Immunol..

[26] Engeman T., Gorbachev A.V., Kish D.D., Fairchild R.L. (2004). The Intensity of Neutrophil Infiltration Controls the Number of Antigen-Primed CD8 T Cells Recruited into Cutaneous Antigen Challenge Sites. J. Leukoc. Biol..

[27] Jee M.H., Funch A.B., Weber J.F., Yeung K., Mraz V., Gadsbøll A.-S.Ø., Song T., Woetmann A., Ødum N., Johansen J.D. (2024). Pre-Existing Inflammation Reduces the Response to Contact Allergens in Tmem79-Deficient Mice. Allergy.

[28] Helou D.G., Noël B., Gaudin F., Groux H., El Ali Z., Pallardy M., Chollet-Martin S., Kerdine-Römer S. (2019). Cutting Edge: Nrf2 Regulates Neutrophil Recruitment and Accumulation in Skin during Contact Hypersensitivity. J. Immunol..

[29] Pesqué D., Andrades E., Berenguer-Molins P., Perera-Bel J., Clarós M., Bódalo-Torruella M., González-Farré M., Gallardo F., Pujol R.M., Giménez-Arnau A.M. (2025). Transcriptomic Analysis of Allergic Patch Test Reactions in Non-Atopic Patients: A Comparative Study Across Multiple Allergens. Allergy.

[30] Basketter D., Dooms-Goossens A., Karlberg A.T., Lepoittevin J.P. (1995). The Chemistry of Contact Allergy: Why Is a Molecule Allergenic?. Contact Dermat..

[31] Funch A.B., Weber J.F., Mraz V., Kongsbak-Wismann M., Lohmann R.K.D., Jee M.H., Vaher H., Yeung K., Gadsbøll A.Ø., Ødum N. (2025). CD8+ Skin-Resident Memory T Cells Require TCR Signaling for Their Persistence in a Mouse Model of Allergic Contact Dermatitis. J. Investig. Dermatol..

[32] Lepoittevin J.P., Leblond I. (1997). Hapten-Peptide-T Cell Receptor Interactions: Molecular Basis for the Recognition of Haptens by T Lymphocytes. Eur. J. Dermatol..

[33] Yeung K., Mraz V., Geisler C., Skov L., Bonefeld C.M. (2021). The Role of Interleukin-1β in the Immune Response to Contact Allergens. Contact Dermat..

[34] Watanabe H., Gaide O., Pétrilli V., Martinon F., Contassot E., Roques S., Kummer J.A., Tschopp J., French L.E. (2007). Activation of the IL-1beta-Processing Inflammasome Is Involved in Contact Hypersensitivity. J. Investig. Dermatol..

[35] Enk A.H., Angeloni V.L., Udey M.C., Katz S.I. (1993). An Essential Role for Langerhans Cell-Derived IL-1 Beta in the Initiation of Primary Immune Responses in Skin. J. Immunol..

[36] Enk A.H., Katz S.I. (1992). Early Molecular Events in the Induction Phase of Contact Sensitivity. Proc. Natl. Acad. Sci. USA.

[37] Antonopoulos C., Cumberbatch M., Mee J.B., Dearman R.J., Wei X.-Q., Liew F.Y., Kimber I., Groves R.W. (2008). IL-18 Is a Key Proximal Mediator of Contact Hypersensitivity and Allergen-Induced Langerhans Cell Migration in Murine Epidermis. J. Leukoc. Biol..

[38] Cumberbatch M., Dearman R.J., Kimber I. (1997). Langerhans Cells Require Signals from Both Tumour Necrosis Factor Alpha and Interleukin 1 Beta for Migration. Adv. Exp. Med. Biol..

[39] Honda T., Nakajima S., Egawa G., Ogasawara K., Malissen B., Miyachi Y., Kabashima K. (2010). Compensatory Role of Langerhans Cells and Langerin-Positive Dermal Dendritic Cells in the Sensitization Phase of Murine Contact Hypersensitivity. J. Allergy Clin. Immunol..

[40] Noordegraaf M., Flacher V., Stoitzner P., Clausen B.E. (2010). Functional Redundancy of Langerhans Cells and Langerin+ Dermal Dendritic Cells in Contact Hypersensitivity. J. Investig. Dermatol..

[41] Martin S., Ortmann B., Pflugfelder U., Birsner U., Weltzien H.U. (1992). Role of Hapten-Anchoring Peptides in Defining Hapten-Epitopes for MHC-Restricted Cytotoxic T Cells. Cross-Reactive TNP-Determinants on Different Peptides. J. Immunol..

[42] Martin S., von Bonin A., Fessler C., Pflugfelder U., Weltzien H.U. (1993). Structural Complexity of Antigenic Determinants for Class I MHC-Restricted, Hapten-Specific T Cells. Two Qualitatively Differing Types of H-2Kb-Restricted TNP Epitopes. J. Immunol..

[43] Lanier L.L., O’Fallon S., Somoza C., Phillips J.H., Linsley P.S., Okumura K., Ito D., Azuma M. (1995). CD80 (B7) and CD86 (B70) Provide Similar Costimulatory Signals for T Cell Proliferation, Cytokine Production, and Generation of CTL. J. Immunol..

[44] Riemann H., Loser K., Beissert S., Fujita M., Schwarz A., Schwarz T., Grabbe S. (2005). IL-12 Breaks Dinitrothiocyanobenzene (DNTB)-Mediated Tolerance and Converts the Tolerogen DNTB into an Immunogen. J. Immunol..

[45] Xu H., DiIulio N.A., Fairchild R.L. (1996). T Cell Populations Primed by Hapten Sensitization in Contact Sensitivity Are Distinguished by Polarized Patterns of Cytokine Production: Interferon Gamma-Producing (Tc1) Effector CD8+ T Cells and Interleukin (Il) 4/Il-10-Producing (Th2) Negative Regulator. J. Exp. Med..

[46] Larsen J.M., Geisler C., Nielsen M.W., Boding L., Von Essen M., Hansen A.K., Skov L., Bonefeld C.M. (2007). Cellular Dynamics in the Draining Lymph Nodes during Sensitization and Elicitation Phases of Contact Hypersensitivity. Contact Dermat..

[47] Vocanson M., Hennino A., Rozières A., Poyet G., Nicolas J.-F. (2009). Effector and Regulatory Mechanisms in Allergic Contact Dermatitis. Allergy.

[48] Smith J.S., Rajagopal S., Atwater A.R. (2018). Chemokine Signaling in Allergic Contact Dermatitis: Toward Targeted Therapies. Dermat. Contact Atopic Occup. Drug.

[49] Flier J., Boorsma D.M., Van Beek P.J., Nieboer C., Stoof T.J., Willemze R., Tensen C.P. (2001). Differential Expression of CXCR3 Targeting Chemokines CXCL10, CXCL9, and CXCL11 in Different Types of Skin Inflammation. J. Pathol..

[50] Flier J., Boorsma D.M., Bruynzeel D.P., Van Beck P.J., Stoof T.J., Scheper R.J., Willemze R., Tensen C.P. (1999). The CXCR3 Activating Chemokines IP-10, Mig, and IP-9 Are Expressed in Allergic but Not in Irritant Patch Test Reactions. J. Investig. Dermatol..

[51] Dudda J.C., Lembo A., Bachtanian E., Huehn J., Siewert C., Hamann A., Kremmer E., Förster R., Martin S.F. (2005). Dendritic Cells Govern Induction and Reprogramming of Polarized Tissue-Selective Homing Receptor Patterns of T Cells: Important Roles for Soluble Factors and Tissue Microenvironments. Eur. J. Immunol..

[52] Zaid A., Hor J.L., Christo S.N., Groom J.R., Heath W.R., Mackay L.K., Mueller S.N. (2017). Chemokine Receptor-Dependent Control of Skin Tissue-Resident Memory T Cell Formation. J. Immunol..

[53] Strzepa A., Gurski C.J., Dittel L.J., Szczepanik M., Pritchard K.A., Dittel B.N. (2020). Neutrophil-Derived Myeloperoxidase Facilitates Both the Induction and Elicitation Phases of Contact Hypersensitivity. Front. Immunol..

[54] Cassatella M.A., Gasperini S., Calzetti F., Bertagnin A., Luster A.D., McDonald P.P. (1997). Regulated Production of the Interferon-γ-Inducible Protein-L0 (IP-10) Chemokine by Human Neutrophils. Eur. J. Immunol..

[55] Jiang X., Park C.O., Geddes Sweeney J., Yoo M.J., Gaide O., Kupper T.S. (2017). Dermal Γδ T Cells Do Not Freely Re-Circulate Out of Skin and Produce IL-17 to Promote Neutrophil Infiltration during Primary Contact Hypersensitivity. PLoS ONE.

[56] Kish D.D., Gorbachev A.V., Fairchild R.L. (2012). IL-1 Receptor Signaling Is Required at Multiple Stages of Sensitization and Elicitation of the Contact Hypersensitivity Response. J. Immunol..

[57] Liu B., Escalera J., Balakrishna S., Fan L., Caceres A.I., Robinson E., Sui A., McKay M.C., McAlexander M.A., Herrick C.A. (2013). TRPA1 Controls Inflammation and Pruritogen Responses in Allergic Contact Dermatitis. FASEB J..

[58] Sakai H., Ishida T., Sato K., Mandokoro K., Yabe S., Sato F., Chiba Y., Kon R., Ikarashi N., Kamei J. (2019). Interference of Skin Scratching Attenuates Accumulation of Neutrophils in Murine Allergic Contact Dermatitis Model. Inflammation.

[59] Oyoshi M.K., He R., Li Y., Mondal S., Yoon J., Afshar R., Chen M., Lee D.M., Luo H.R., Luster A.D. (2012). Leukotriene B4-Driven Neutrophil Recruitment to the Skin Is Essential for Allergic Skin Inflammation. Immunity.

[60] Yang Y., Pan Y., Liu B., Zhang Y., Yin C., Wang J., Nie H., Xu R., Tai Y., He X. (2024). Neutrophil-Derived Oxidative Stress Contributes to Skin Inflammation and Scratching in a Mouse Model of Allergic Contact Dermatitis via Triggering pro-Inflammatory Cytokine and Pruritogen Production in Skin. Biochem. Pharmacol..

[61] Arnason B.G., Waksman B.H. (1963). The Retest Reaction in Delayed Sensitivity. Lab. Investig..

[62] Nakagawa S., Fukushiro S., Gotoh M., Kohda M., Namba M., Tanioku K. (1978). Studies on the Retest Reaction in Contact Sensitivity to DNCB. Dermatologica.

[63] Scheper R.J., von Blomberg M., Boerrigter G.H., Bruynzeel D., van Dinther A., Vos A. (1983). Induction of Immunological Memory in the Skin. Role of Local T Cell Retention. Clin. Exp. Immunol..

[64] Gebhardt T., Wakim L.M., Eidsmo L., Reading P.C., Heath W.R., Carbone F.R. (2009). Memory T Cells in Nonlymphoid Tissue That Provide Enhanced Local Immunity during Infection with Herpes Simplex Virus. Nat. Immunol..

[65] Gaide O., Emerson R.O., Jiang X., Gulati N., Nizza S., Desmarais C., Robins H., Krueger J.G., Clark R.A., Kupper T.S. (2015). Common Clonal Origin of Central and Resident Memory T Cells Following Skin Immunization. Nat. Med..

[66] Gadsbøll A.-S.Ø., Jee M.H., Funch A.B., Alhede M., Mraz V., Weber J.F., Callender L.A., Carroll E.C., Bjarnsholt T., Woetmann A. (2020). Pathogenic CD8+ Epidermis-Resident Memory T Cells Displace Dendritic Epidermal T Cells in Allergic Dermatitis. J. Investig. Dermatol..

[67] Sallusto F., Lenig D., Förster R., Lipp M., Lanzavecchia A. (1999). Two Subsets of Memory T Lymphocytes with Distinct Homing Potentials and Effector Functions. Nature.

[68] Sallusto F., Geginat J., Lanzavecchia A. (2004). Central Memory and Effector Memory T Cell Subsets: Function, Generation, and Maintenance. Annu. Rev. Immunol..

[69] Karlberg A.T., Bergström M.A., Börje A., Luthman K., Nilsson J.L.G. (2008). Allergic Contact Dermatitis—Formation, Structural Requirements, and Reactivity of Skin Sensitizers. Chem. Res. Toxicol..

[70] Gamradt P., Laoubi L., Nosbaum A., Mutez V., Lenief V., Grande S., Redoulès D., Schmitt A.-M., Nicolas J.-F., Vocanson M. (2019). Inhibitory Checkpoint Receptors Control CD8+ Resident Memory T Cells to Prevent Skin Allergy. J. Allergy Clin. Immunol..

[71] He D., Wu L., Kim H.K., Li H., Elmets C.A., Xu H. (2009). IL-17 and IFN-Gamma Mediate the Elicitation of Contact Hypersensitivity Responses by Different Mechanisms and Both Are Required for Optimal Responses. J. Immunol..

[72] Albanesi C., Cavani A., Girolomoni G. (1998). Interferon-Gamma-Stimulated Human Keratinocytes Express the Genes Necessary for the Production of Peptide-Loaded MHC Class II Molecules. J. Investig. Dermatol..

[73] Trautmann A., Akdis M., Kleemann D., Altznauer F., Simon H.U., Graeve T., Noll M., Bröcker E.B., Blaser K., Akdis C.A. (2000). T Cell-Mediated Fas-Induced Keratinocyte Apoptosis Plays a Key Pathogenetic Role in Eczematous Dermatitis. J. Clin. Investig..

[74] Chong S.Z., Tan K.W., Wong F.H.S., Chua Y.L., Tang Y., Ng L.G., Angeli V., Kemeny D.M. (2014). CD8 T Cells Regulate Allergic Contact Dermatitis by Modulating CCR2-Dependent TNF/INOS-Expressing Ly6C+ CD11b+ Monocytic Cells. J. Investig. Dermatol..

[75] Kish D.D., Li X., Fairchild R.L. (2009). CD8 T Cells Producing IL-17 and IFN-γ Initiate the Innate Immune Response Required for Responses to Antigen Skin Challenge. J. Immunol..

[76] Kish D.D., Volokh N., Baldwin W.M., Fairchild R.L. (2011). Hapten Application to the Skin Induces an Inflammatory Program Directing Hapten-Primed Effector CD8 T Cell Interaction with Hapten-Presenting Endothelial Cells. J. Immunol..

[77] Kursawe Larsen C., Funch A.B., Vaher H., Lohmann R.K.D., Jee M.H., Schwensen J.F.B., Zachariae C., Svedman C., Bergendorff O., Bonefeld C.M. (2024). Cross-Reactivity between Thiuram Disulfides and Dithiocarbamates. A Study of TETD and ZDEC Using Mouse Models. Contact Dermat..

[78] Hasegawa Y., Iwata Y., Fukushima H., Tanaka Y., Watanabe S., Saito K., Ito H., Sugiura M., Akiyama M., Sugiura K. (2022). Neutrophil Extracellular Traps Are Involved in Enhanced Contact Hypersensitivity Response in IL-36 Receptor Antagonist-Deficient Mice. Sci. Rep..

[79] Azeem M., Helal M., Klein-Hessling S., Serfling E., Goebeler M., Muhammad K., Kerstan A. (2025). NFATc1 Fosters Allergic Contact Dermatitis Responses by Enhancing the Induction of IL-17-Producing CD8 Cells. J. Investig. Dermatol..

[80] Watanabe H., Gehrke S., Contassot E., Roques S., Tschopp J., Friedmann P.S., French L.E., Gaide O. (2008). Danger Signaling through the Inflammasome Acts as a Master Switch between Tolerance and Sensitization. J. Immunol..

[81] Hirai T., Yang Y., Zenke Y., Li H., Chaudhri V.K., De La Cruz Diaz J.S., Zhou P.Y., Nguyen B.A.-T., Bartholin L., Workman C.J. (2021). Competition for Active TGFβ Cytokine Allows for Selective Retention of Antigen-Specific Tissue- Resident Memory T Cells in the Epidermal Niche. Immunity.

[82] Adachi T., Kobayashi T., Sugihara E., Yamada T., Ikuta K., Pittaluga S., Saya H., Amagai M., Nagao K. (2015). Hair Follicle-Derived IL-7 and IL-15 Mediate Skin-Resident Memory T Cell Homeostasis and Lymphoma. Nat. Med..

[83] Mackay L.K., Wynne-Jones E., Freestone D., Pellicci D.G., Mielke L.A., Newman D.M., Braun A., Masson F., Kallies A., Belz G.T. (2015). T-Box Transcription Factors Combine with the Cytokines TGF-β and IL-15 to Control Tissue-Resident Memory T Cell Fate. Immunity.

[84] Christo S.N., Evrard M., Park S.L., Gandolfo L.C., Burn T.N., Fonseca R., Newman D.M., Alexandre Y.O., Collins N., Zamudio N.M. (2021). Discrete Tissue Microenvironments Instruct Diversity in Resident Memory T Cell Function and Plasticity. Nat. Immunol..

[85] Cheuk S., Schlums H., Gallais Sérézal I., Martini E., Chiang S.C., Marquardt N., Gibbs A., Detlofsson E., Introini A., Forkel M. (2017). CD49a Expression Defines Tissue-Resident CD8+ T Cells Poised for Cytotoxic Function in Human Skin. Immunity.

[86] Richmond J.M., Strassner J.P., Zapata L., Garg M., Riding R.L., Refat M.A., Fan X., Azzolino V., Tovar-Garza A., Tsurushita N. (2018). Antibody Blockade of IL-15 Signaling Has the Potential to Durably Reverse Vitiligo. Sci. Transl. Med..

[87] Xing L., Dai Z., Jabbari A., Cerise J.E., Higgins C.A., Gong W., De Jong A., Harel S., Destefano G.M., Rothman L. (2014). Alopecia Areata Is Driven by Cytotoxic T Lymphocytes and Is Reversed by JAK Inhibition. Nat. Med..

[88] Evrard M., Wynne-Jones E., Peng C., Kato Y., Christo S.N., Fonseca R., Park S.L., Burn T.N., Osman M., Devi S. (2022). Sphingosine 1-Phosphate Receptor 5 (S1PR5) Regulates the Peripheral Retention of Tissue-Resident Lymphocytes. J. Exp. Med..

[89] Mora-Buch R., Lake M.E., Sama A., Chasse A.Y., Akbaba H., Mani V., Bromley S.K. (2025). IL-4 Impairs the Formation of Skin-Resident Memory CD8^+^ T Cells. Nat. Immunol..

[90] Mackay L.K., Stock A.T., Ma J.Z., Jones C.M., Kent S.J., Mueller S.N., Heath W.R., Carbone F.R., Gebhardt T. (2012). Long-Lived Epithelial Immunity by Tissue-Resident Memory T (TRM) Cells in the Absence of Persisting Local Antigen Presentation. Proc. Natl. Acad. Sci. USA.

[91] Abdelbary M., Hobbs S.J., Gibbs J.S., Yewdell J.W., Nolz J.C. (2023). T Cell Receptor Signaling Strength Establishes the Chemotactic Properties of Effector CD8+ T Cells That Control Tissue-Residency. Nat. Commun..

[92] Khan T.N., Mooster J.L., Kilgore A.M., Osborn J.F., Nolz J.C. (2016). Local Antigen in Nonlymphoid Tissue Promotes Resident Memory CD8+ T Cell Formation during Viral Infection. J. Exp. Med..

[93] Muschaweckh A., Buchholz V.R., Fellenzer A., Hessel C., König P.-A., Tao S., Tao R., Heikenwälder M., Busch D.H., Korn T. (2016). Antigen-Dependent Competition Shapes the Local Repertoire of Tissue-Resident Memory CD8+ T Cells. J. Exp. Med..

[94] Jacob S.E., Castanedo-Tardan M.P. (2007). Pharmacotherapy for Allergic Contact Dermatitis. Expert Opin. Pharmacother..

[95] Ono E., Lenief V., Lefevre M.-A., Cuzin R., Guironnet-Paquet A., Mosnier A., Nosbaum A., Nicolas J.-F., Vocanson M. (2024). Topical Corticosteroids Inhibit Allergic Skin Inflammation but Are Ineffective in Impeding the Formation and Expansion of Resident Memory T Cells. Allergy.

[96] Natsuaki Y., Egawa G., Nakamizo S., Ono S., Hanakawa S., Okada T., Kusuba N., Otsuka A., Kitoh A., Honda T. (2014). Perivascular Leukocyte Clusters Are Essential for Efficient Activation of Effector T Cells in the Skin. Nat. Immunol..

[97] Goksøyr L., Funch A.B., Okholm A.K., Theander T.G., de Jongh W.A., Bonefeld C.M., Sander A.F. (2022). Preclinical Efficacy of a Capsid Virus-like Particle-Based Vaccine Targeting IL-1β for Treatment of Allergic Contact Dermatitis. Vaccines.

[98] Sitaru S., Budke A., Bertini R., Sperandio M. (2023). Therapeutic Inhibition of CXCR1/2: Where Do We Stand?. Intern. Emerg. Med..

[99] Jurcevic S., Humfrey C., Uddin M., Warrington S., Larsson B., Keen C. (2015). The Effect of a Selective CXCR2 Antagonist (AZD5069) on Human Blood Neutrophil Count and Innate Immune Functions. Br. J. Clin. Pharmacol..

[100] Rennard S.I., Dale D.C., Donohue J.F., Kanniess F., Magnussen H., Sutherland E.R., Watz H., Lu S., Stryszak P., Rosenberg E. (2015). CXCR2 Antagonist MK-7123. A Phase 2 Proof-of-Concept Trial for Chronic Obstructive Pulmonary Disease. Am. J. Respir. Crit. Care Med..

[101] Kirsten A.M., Förster K., Radeczky E., Linnhoff A., Balint B., Watz H., Wray H., Salkeld L., Cullberg M., Larsson B. (2015). The Safety and Tolerability of Oral AZD5069, a Selective CXCR2 Antagonist, in Patients with Moderate-to-Severe COPD. Pulm. Pharmacol. Ther..

[102] O’Byrne P.M., Metev H., Puu M., Richter K., Keen C., Uddin M., Larsson B., Cullberg M., Nair P. (2016). Efficacy and Safety of a CXCR2 Antagonist, AZD5069, in Patients with Uncontrolled Persistent Asthma: A Randomised, Double-Blind, Placebo-Controlled Trial. Lancet Respir. Med..

[103] Blair H.A. (2021). Secukinumab: A Review in Psoriatic Arthritis. Drugs.

[104] Al-Salama Z.T. (2019). Emapalumab: First Global Approval. Drugs.

[105] Leonardi C., Matheson R., Zachariae C., Cameron G., Li L., Edson-Heredia E., Braun D., Banerjee S. (2012). Anti–Interleukin-17 Monoclonal Antibody Ixekizumab in Chronic Plaque Psoriasis. N. Engl. J. Med..

[106] Wu D., Hou S.-Y., Zhao S., Hou L.-X., Jiao T., Xu N.-N., Zhang N. (2017). Efficacy and Safety of Interleukin-17 Antagonists in Patients with Plaque Psoriasis: A Meta-Analysis from Phase 3 Randomized Controlled Trials. J. Eur. Acad. Dermatol. Venereol..

[107] Ly K., Smith M.P., Thibodeaux Q., Reddy V., Liao W., Bhutani T. (2019). Anti IL-17 in Psoriasis. Expert Rev. Clin. Immunol..

[108] Todberg T., Zachariae C., Krustrup D., Skov L. (2019). The Effect of Anti-IL-17 Treatment on the Reaction to a Nickel Patch Test in Patients with Allergic Contact Dermatitis. Int. J. Dermatol..

[109] Feng Y., Zhou B., Wang Z., Xu G., Wang L., Zhang T., Zhang Y. (2022). Risk of Candida Infection and Serious Infections in Patients with Moderate-to-Severe Psoriasis Receiving Biologics: A Systematic Review and Meta-Analysis of Randomized Controlled Trials. Int. J. Clin. Pract..

[110] Liu H., Zhou L., Song Z., Zhang R., Kang Y. (2025). Biologic Therapy and Superficial Fungal Infection Risk in Moderate-to-Severe Psoriasis: A Meta-Analysis. Mycoses.

[111] Qi F., Liu F., Gao L. (2021). Janus Kinase Inhibitors in the Treatment of Vitiligo: A Review. Front. Immunol..

[112] Pratt C.H., King L.E., Messenger A.G., Christiano A.M., Sundberg J.P. (2017). Alopecia Areata. Nat. Rev. Dis. Prim..

[113] Liu M., Gao Y., Yuan Y., Yang K., Shen C., Wang J., Tian J. (2023). Janus Kinase Inhibitors for Alopecia Areata: A Systematic Review and Meta-Analysis. JAMA Netw. Open.

[114] Traidl S., Freimooser S., Werfel T. (2021). Janus Kinase Inhibitors for the Therapy of Atopic Dermatitis. Allergol. Sel..

[115] Ingrassia J.P., Maqsood M.H., Gelfand J.M., Weber B.N., Bangalore S., Lo Sicco K.I., Garshick M.S. (2024). Cardiovascular and Venous Thromboembolic Risk With JAK Inhibitors in Immune-Mediated Inflammatory Skin Diseases: A Systematic Review and Meta-Analysis. JAMA Dermatol..

[116] Isufi D., Jensen M.B., Loft N., Skov L., Elberling J., Alinaghi F. (2025). Risk of Infections during Treatment with Oral Janus Kinase Inhibitors in Randomized Placebo-Controlled Trials: A Systematic Review and Meta-Analysis. JAAD Int..

[117] Tieu R., Zeng Q., Zhao D., Zhang G., Feizi N., Manandhar P., Williams A.L., Popp B., Wood-Trageser M.A., Demetris A.J. (2023). Tissue-Resident Memory T Cell Maintenance during Antigen Persistence Requires Both Cognate Antigen and Interleukin-15. Sci. Immunol..

[118] Teicher B.A. (2021). TGFβ-Directed Therapeutics: 2020. Pharmacol. Ther..

[119] Keir M.E., Butte M.J., Freeman G.J., Sharpe A.H. (2008). PD-1 and Its Ligands in Tolerance and Immunity. Annu. Rev. Immunol..

[120] Biemond M., Vremec D., Gray D.H.D., Hodgkin P.D., Heinzel S. (2024). Programmed Death Receptor 1 (PD-1) Ligand Fc Fusion Proteins Reduce T-Cell Proliferation in Vitro Independently of PD-1. Immunol. Cell Biol..

[121] Tetsu H., Nakayama K., Nishijo T., Yuki T., Miyazawa M. (2023). CTLA-4 Suppresses Hapten-Induced Contact Hypersensitivity in Atopic Dermatitis Model Mice. Sci. Rep..

[122] Dean J.W., Helm E.Y., Fu Z., Xiong L., Sun N., Oliff K.N., Muehlbauer M., Avram D., Zhou L. (2023). The Aryl Hydrocarbon Receptor Cell Intrinsically Promotes Resident Memory CD8+ T Cell Differentiation and Function. Cell Rep..

[123] Correia M.P., Jeong M., Ast V., Platten M., Sexl V., Mogler C. (2022). AhR-Mediated Activation of Innate Lymphocytes Restrains Tissue-Resident Memory-like CD8 + T Cell Responses during Contact Hypersensitivity. bioRxiv.

[124] Chi H. (2012). Regulation and Function of MTOR Signalling in T Cell Fate Decisions. Nat. Rev. Immunol..

[125] Smith S.H., Jayawickreme C., Rickard D.J., Nicodeme E., Bui T., Simmons C., Coquery C.M., Neil J., Pryor W.M., Mayhew D. (2017). Tapinarof Is a Natural AhR Agonist That Resolves Skin Inflammation in Mice and Humans. J. Investig. Dermatol..

[126] Bobonich M., Gorelick J., Aldredge L., Bruno M.J., DiRuggiero D., Martin G., Tallman A.M., Gold L.S. (2023). Tapinarof, a Novel, First-in-Class, Topical Therapeutic Aryl Hydrocarbon Receptor Agonist for the Management of Psoriasis. J. Drugs Dermatol..

[127] Lebwohl M.G., Stein Gold L., Strober B., Papp K.A., Armstrong A.W., Bagel J., Kircik L., Ehst B., Hong H.C.-H., Soung J. (2021). Phase 3 Trials of Tapinarof Cream for Plaque Psoriasis. N. Engl. J. Med..

[128] Bissonnette R., Stein Gold L., Rubenstein D.S., Tallman A.M., Armstrong A. (2021). Tapinarof in the Treatment of Psoriasis: A Review of the Unique Mechanism of Action of a Novel Therapeutic Aryl Hydrocarbon Receptor-Modulating Agent. J. Am. Acad. Dermatol..

[129] Alexis A.F., Kircik L., Chovatiya R., Rice Z.P., Soong W., Bhutani T., Brown P.M., Piscitelli S.C., Rubenstein D.S., Tallman A.M. (2025). Tapinarof Cream for Adults and Children with Atopic Dermatitis-Efficacy by Race and Fitzpatrick Skin Type in Two Phase 3 Randomized Clinical Trials. Dermatol. Ther..

[130] Simpson E.L., Hebert A.A., Browning J., Serrao R.T., Sofen H., Brown P.M., Piscitelli S.C., Rubenstein D.S., Tallman A.M. (2025). Tapinarof Improved Outcomes and Sleep for Patients and Families in Two Phase 3 Atopic Dermatitis Trials in Adults and Children. Dermatol. Ther..

[131] Hou H., Miao J., Cao R., Han M., Sun Y., Liu X., Guo L. (2017). Rapamycin Ameliorates Experimental Autoimmune Encephalomyelitis by Suppressing the MTOR-STAT3 Pathway. Neurochem. Res..

[132] Esposito M., Ruffini F., Bellone M., Gagliani N., Battaglia M., Martino G., Furlan R. (2010). Rapamycin Inhibits Relapsing Experimental Autoimmune Encephalomyelitis by Both Effector and Regulatory T Cells Modulation. J. Neuroimmunol..

[133] Fernandez D., Bonilla E., Mirza N., Niland B., Perl A. (2006). Rapamycin Reduces Disease Activity and Normalizes T Cell Activation-Induced Calcium Fluxing in Patients with Systemic Lupus Erythematosus. Arthritis Rheum..

[134] Geier C., Perl A. (2021). Therapeutic MTOR Blockade in Systemic Autoimmunity: Implications for Antiviral Immunity and Extension of Lifespan. Autoimmun. Rev..

[135] Capella G.L., Grigerio E., Altomare G. (2001). A Randomized Trial of Leukotriene Receptor Antagonist Montelukast in Moderate-to-Severe Atopic Dermatitis of Adults. Eur. J. Dermatol..

[136] Veien N.K., Busch-Sørensen M., Stausbøl-Grøn B. (2005). Montelukast Treatment of Moderate to Severe Atopic Dermatitis in Adults: A Randomized, Double-Blind, Placebo-Controlled Trial. J. Am. Acad. Dermatol..

[137] Heber S., Gold-Binder M., Ciotu C.I., Witek M., Ninidze N., Kress H.-G., Fischer M.J.M. (2019). A Human TRPA1-Specific Pain Model. J. Neurosci..

[138] Chen Y.-L., Gutowska-Owsiak D., Hardman C.S., Westmoreland M., MacKenzie T., Cifuentes L., Waithe D., Lloyd-Lavery A., Marquette A., Londei M. (2019). Proof-of-Concept Clinical Trial of Etokimab Shows a Key Role for IL-33 in Atopic Dermatitis Pathogenesis. Sci. Transl. Med..

[139] Silverberg J.I., Mustapa M.N., Reid F., Lei A., Smith R., Moate R., Kelly A., Chen R., Gavala M., Jimenez E. (2025). Efficacy and Safety of Tozorakimab in Moderate-to-Severe Atopic Dermatitis: A Phase 2a Randomized Controlled Trial (FRONTIER-2). J. Eur. Acad. Dermatol. Venereol..

[140] Zuberbier T., Aberer W., Asero R., Abdul Latiff A.H., Baker D., Ballmer-Weber B., Bernstein J.A., Bindslev-Jensen C., Brzoza Z., Buense Bedrikow R. (2018). The EAACI/GA^2^LEN/EDF/WAO Guideline for the Definition, Classification, Diagnosis and Management of Urticaria. Allergy.

[141] Church M.K., Church D.S. (2013). Pharmacology of Antihistamines. Indian J. Dermatol..

[142] Naicker P., Anoopkumar-Dukie S., Grant G.D., Kavanagh J.J. (2013). The Effects of Antihistamines with Varying Anticholinergic Properties on Voluntary and Involuntary Movement. Clin. Neurophysiol..

[143] van Ruissen M.C.E., van Kraaij S.J.W., Wolfova J., Herrmann F.E., Botilde Y., Wollin L., Klarenbeek N.B. (2025). Proof of Pharmacology, Safety, and Pharmacokinetics of the Novel TRPA1 Antagonist BI 1839100: A Randomized, Placebo-Controlled, Parallel Group, First-In-Human Study in Healthy Male Participants. Clin. Transl. Sci..

[144] Lepoittevin J.-P. (2006). Metabolism versus Chemical Transformation or Pro- versus Prehaptens?. Contact Dermat..

[145] El Ali Z., Gerbeix C., Hemon P., Esser P.R., Martin S.F., Pallardy M., Kerdine-Römer S. (2013). Allergic Skin Inflammation Induced by Chemical Sensitizers Is Controlled by the Transcription Factor Nrf_2_. Toxicol. Sci..

[146] Houghton C.A., Fassett R.G., Coombes J.S. (2016). Sulforaphane and Other Nutrigenomic Nrf_2_ Activators: Can the Clinician’s Expectation Be Matched by the Reality?. Oxid. Med. Cell. Longev..

[147] Rice L.M., Padilla C.M., McLaughlin S.R., Mathes A., Ziemek J., Goummih S., Nakerakanti S., York M., Farina G., Whitfield M.L. (2015). Fresolimumab Treatment Decreases Biomarkers and Improves Clinical Symptoms in Systemic Sclerosis Patients. J. Clin. Investig..

[148] Balato A., Lembo S., Ayala F., Balato N., Caiazzo G., Raimondo A., Di Caprio R., Monfrecola G. (2017). Mechanistic Target of Rapamycin Complex 1 Is Involved in Psoriasis and Regulated by Anti-TNF-α Treatment. Exp. Dermatol..

